# Neural correlates of lexical-semantic memory: A voxel-based morphometry study in mild AD, aMCI and normal aging

**DOI:** 10.1590/S1980-57642011DN05020003

**Published:** 2011

**Authors:** Marcio L.F. Balthazar, Clarissa L. Yasuda, Tátila M. Lopes, Fabrício R.S. Pereira, Benito Pereira Damasceno, Fernando Cendes

**Affiliations:** 1 Laboratory of Neuroimaging, Department of Neurology, Medical School, University of Campinas (UNICAMP), Campinas SP, Brazil.

**Keywords:** semantic memory, naming, voxel-based morphometry, Alzheimer’s disease, mild cognitive impairment

## Abstract

Neuroanatomical correlations of naming and lexical-semantic memory are not yet fully understood. The most influential approaches share the view that semantic representations reflect the manner in which information has been acquired through perception and action, and that each brain area processes different modalities of semantic representations. Despite these anatomical differences in semantic processing, generalization across different features that have similar semantic significance is one of the main characteristics of human cognition.

**Methods:**

We evaluated the brain regions related to naming, and to the semantic generalization, of visually presented drawings of objects from the Boston Naming Test (BNT), which comprises different categories, such as animals, vegetables, tools, food, and furniture. In order to create a model of lesion method, a sample of 48 subjects presenting with a continuous decline both in cognitive functions, including naming skills, and in grey matter density (GMD) was compared to normal young adults with normal aging, amnestic mild cognitive impairment (aMCI) and mild Alzheimer’s disease (AD). Semantic errors on the BNT, as well as naming performance, were correlated with whole brain GMD as measured by voxel-based morphometry (VBM).

**Results:**

The areas most strongly related to naming and to semantic errors were the medial temporal structures, thalami, superior and inferior temporal gyri, especially their anterior parts, as well as prefrontal cortices (inferior and superior frontal gyri).

**Conclusion:**

The possible role of each of these areas in the lexical-semantic networks was discussed, along with their contribution to the models of semantic memory organization.

## Abstract (pt)

As correlações neuroanatômicas de nomeação e memória lexical-semântica não são totalmente entendidas. As abordagens mais influentes compartilham a idéia de que representações refletem a maneira na qual a informação foi adquirida por meio da percepção e ação e que cada área do cérebro processa diferentes modalidades de representações semânticas. Apesar destas diferenças anatômicas no processamento semântico, a generalização de diferentes aspectos que tem significância semântica análoga é uma das principais características da cognição humana.

**Métodos:**

Nós avaliamos as regiões cerebrais relacionadas à nomeação e à generalização semântica de desenhos de objetos visualmente apresentados do Teste de Nomeação de Boston (TNB), que compreende diferentes categorias, como animais, vegetais, utensílios, comida e móveis. Para criar um modelo de método de lesão, nós investigamos uma amostra de 48 pessoas que demonstraram um declínio contínuo em funções cognitivas, incluindo habilidades de nomeação e em densidade de substância cinzenta (DSC) em relação a adultos jovens: envelhecimento normal, comprometimento cognitivo leve amnéstico (CCLa) e doença de Alzheimer (DA). Nós correlacionamos seus erros semânticos no TNB, assim como seus desempenhos de nomeação, com a DSC de todo o cérebro que foi medido por morfometria baseada em vóxel (MBV).

**Resultados:**

As áreas que mais se relacionaram à nomeação e aos erros semânticos foram as estruturas mediais temporais, tálamo, giro temporal superior e inferior, especialmente em suas partes anteriores e os córtices pré-frontais (giro frontal inferior e superior).

**Conclusão:**

Discutimos o provável papel de cada uma dessas áreas na rede lexical-semântica e sua contribuição para os modelos de organização semântica.

Language is one of the most important characteristics that allows us to codify, signify, and retain our experience of the world.^1^ Naming the many aspects of our environment is an essential attribute for the evolution of human complex adaptive ability and reveals the capacity to learn and share knowledge. Lexical-semantic memory refers to the storage of this knowledge in the brain by means of patterns of neuronal activity interpreted as linguistic symbols of concrete and abstract concepts. The relationship between brain anatomy and the storage of these patterns of information is not yet well understood. Several hypotheses have been proposed to explain how lexical-semantic memory is processed and stored in the brain, and these have been guided by two main general models: a parallel distributed representation,^2^ comprising a homogeneous network of equivalent neuronal units that process every aspect of semantics, and a center processing model, which assumes that all memory elements are encoded in a delimited area of the brain. Neither of these models in its pure form satisfactorily explains the phenomena, and so a combination of these two theories has been proposed.^3^

One of the main characteristics of human cognition is the capacity to generalize across concepts that have similar semantic significance but not necessarily similar specific (physical or behavioural) attributes. The most striking evidence of deterioration of this generalizing capacity, manifested initially by semantic naming errors production, is semantic dementia (SD), in which there is a degeneration of the anterior portions of the temporal lobes, and is more intense on the left side. These patients have difficulties in naming everyday objects and knowing their properties, with impairment of all kinds of concepts in the context of otherwise well-preserved cognition, including episodic memory. Other diseases associated with lesions in the anterior parts of temporal lobe show the same pattern of loss of knowledge, particularly in Herpes simplex virus encephalitis, stroke, and Alzheimer’s disease (AD). In this sense, as proposed by other authors, the temporal lobe, particularly its anterior part, may constitute a convergence zone for information coming from brain regions responsible for processing different aspects of knowledge.^4,5^ It has also been suggested that the temporal lobe object representation system may be organized hierarchically, with increasing convergence and integration of information occurring along its posterior to anterior axis.^6^

Naming complaints are very common in mentally healthy elderly people. Individuals over the age of seventy attain significantly lower scores on these naming tests compared to scores by young adults.^7-9^ Problems with naming and word finding are even more common in mild cognitive impairment (MCI) and are most common in Alzheimer’s disease (AD).^10,11^ MCI is a clinical entity applied to patients with objective cognitive problems, most commonly in episodic memory, without significant impairment of activities of daily life.^12^

Our aims were to evaluate the brain regions related to naming performance and to spontaneous semantic naming errors on the Boston Naming Test (BNT),^13^ regardless of category (animals, vegetables, tools, food, and furniture, etc). In order to create a model of the lesion method, a sample of subjects presenting with continuous decline both in cognitive functions, including naming skills, and in grey matter density (GMD) were compared to normal young adults with normal aging, amnestic mild cognitive impairment (aMCI), and mild AD. Semantic errors on the BNT were correlated with whole GMD as measured by voxel-based morphometry (VBM). This correlation was also performed for BNT total score (correct responses). We hypothesised that temporal lobes, especially their anterior parts, are related with semantic naming error production in this sample of subjects. The majority of structural neuroimaging studies in patients with language problems have employed volumetric measurements on magnetic resonance imaging (MRI) data sets. This kind of approach has used the region-of-interest method, which depends on a priori choices and can be applied to a selected set of brain structures only. A whole-brain VBM approach on the other hand, has the advantage of not only evaluating the previously hypothesized brain structures, but also potentially revealing unexpected areas of gray matter density changes and their correlation with neuropsychological scores.

## Methods

A total of 48 subjects older than 50 years [17=aMCI, 15=mild AD treated at the Unit for Neuropsychology and Neurolinguistics (UNICAMP Clinic Hospital), and 16=controls] were studied. Routine laboratory examinations for dementia assessment (including B12 and folate levels, serology for syphilis, and thyroid hormone measurement) and brain computed tomography were carried out in all patients. The local ethics committee approved this study. Diagnosis of aMCI in our clinic is carried out by trained neurologists using a standardized mental state battery, including evaluation of episodic memory, orientation, language, attention, abstract thinking, calculation, and visual perception. The diagnostic process consists of a detailed interview with the patient and informant (usually a close relative of the patient). Diagnosis of MCI was made according to the criteria of the International Working Group on Mild Cognitive Impairment:^13^

(i) the person is neither normal nor demented;
(ii) there is evidence of cognitive deterioration shown by either objectively measured decline over time and/or subjective report of decline by self and/or informant in conjunction with objective cognitive deficits; and
(iii) activities of daily living are preserved and complex instrumental functions are either intact or minimally impaired.

A diagnosis of aMCI was determined if the clinical history and cognitive performance pointed to an exclusive memory deficit and Clinical Dementia Rating (CDR)^14^ score of 0.5, with an obligatory and exclusive memory score of 0.5. This classification was performed using a semi-structured interview.

For probable AD diagnosis, the criteria of the National Institute of Neurological and Communicative Disorders and Stroke (NINCDS) and Alzheimer’s Disease and Related Disorders Association (ADRDA) were employed,^15^ including only patients classified as CDR 1. Exclusion criteria were history of other neurological or psychiatric diseases, head injury with loss of consciousness, use of sedative drugs in the 24 hours preceding the neuropsychological assessment, drug or alcohol addiction, and prior chronic exposure to neurotoxic substances. The control group comprised subjects with CDR 0 and no previous history of neurological or psychiatric disease, or memory complaints.

### Assessment of naming ability

The sixty-item BNT^13^ (translated and culturally adapted version for the Brazilian population by Dr. Cândida Camargo - Psychiatry Institute, University of São Paulo School of Medicine), for which subjects were asked to name the presented pictures, was administered to all subjects. BNT total score was calculated by adding the number of correct spontaneous responses to the number of correct responses after a semantic cue, which consisted of a short explanation about the picture (for example, for mask: it’s part of a carnival costume) or a superordinate category (e.g. for elephant: it’s a kind of animal). The semantic cue was only given if the patient had failed to recognize the picture (for example: dog instead of tree) or if he/she said that they did not know what the picture was.

Semantic errors registered when the spontaneous answer was semantically related to the target word. Two independent researchers performed this classification, and the discordances were solved by consensus.

### Additional neuropsychological evaluation

All subjects were submitted to tests of verbal fluency (VF) - animals category (score=total number of different animal names/one minute); Mini Mental State Examination^16^ (Brazilian version);^17^ Rey auditory verbal learning test^18^- episodic memory delayed recall (RAVLT-A7) and CAMCOG’s subscale of similarities [pairs of nouns - “In what way are they alike?” for the following pairs: apple/banana, chair/table, shirt/dress, and animal/vegetable (score= correct number responses- zero to two for each pair; maximum score eight)];^19^ visual perception subtests of Luria’s Neuropsychological Investigation (LNI; maximum score twenty);^20^ the forward (FDS) and backward digit span (BDS) subtests of the WAIS-R^21^ and the Cornell Scale for Depression in Dementia (CSDD).^22,23^ Data analysis was performed using Systat software 12.0. The Kruskall-Wallis and Mann-Whitney tests were used for inter-group comparisons of demographic and cognitive scores. Statistical significance was considered when p<0.05.

### MRI scanning protocol and imaging processing

High-resolution MRI was performed using a 2.0 T scanner (Elscint, Haifa, Israel). T1- and T2-weighted images were acquired in axial, coronal and sagittal planes with thin slices. In addition, volumetric (3D) T1 gradient echo (GRE) images were acquired in the sagittal plane with 1 mm-thick slices (flip angle=35º, time to repeat=22 ms, echo time=9 ms, matrix=256×220, field of view=23×25 cm). Before pre-processing all scans were checked for scanner artefacts and gross anatomical abnormalities. MRIcro was used to convert the original DICOM format to ANALYZE format (www.mricro.com) and set the origin of the coordinate system at the anterior commissure.

SPM8b (Wellcome Department of Imaging Neuroscience, London, England; www.fil.ion.ucl.ac.uk) run on MATLAB 7.5 was used to perform voxel-based morphometry (VBM). For segmentation, the “New Segment” toolbox from SPM8b was employed. The algorithm used for segmentation is based on “Unified Segmentation”,^24^ which in turn is based on a mixture of Gaussian and combines image registration, tissue classification and bias correction within one generative model. In order to obtain a more accurate inter-subject alignment the DARTEL (Diffeomorphic Anatomical Registration Through Exponential Lie Algebra) registration model was chosen. In addition, to preserve the volume of each tissue (modulation step) the warped images were scaled using the Jacobian determinants. Finally, the normalized, segmented, modulated (Jacobian-corrected) and warped images were smoothed by convolving with an isotropic Gaussian kernel with full width at half maximum of 10 mm to reduce interindividual gyral variation.^25^ After this pre-processing, the resulting normalized, modulated and smoothed data was used for statistics treatment.

### Voxel-based correlation analysis

Multiple regression analysis using Non-Parametric Mapping (NPM) software (http://www.sph.sc.edu/comd/rorden/npm) to identify brain regions whose GMD values were significantly correlated with the absolute number of semantic errors and BNT total score. Age, education, total intracranial volume and global cognition (as measured by MMSE) were also included in the analysis as dependent variables. Total intracranial volume was obtained by the sum of volumes of grey matter, white matter and cerebrospinal fluid. For multiple regression analysis, the three groups were considered together (normal aging, aMCI and mild AD) to increase data variance and enhance the correlation between cerebral region and psychological function, assuming that the denser the gray matter, the better the naming performance and vice versa.

The results were corrected for multiple comparisons by using Bonferroni Correction, which involves adjustments to the statistical threshold to control for overall familywise error rate (FWE). To control for FWE, permutation analysis was also performed using NPM, with 4000 permutations analyzed.^26^

### RESULTS

As shown in Table 1, no significant difference was found among the three groups with regard to age (p=0.17) or education (p=0.31). There was a continuum in neuropsychological performance on all tests, except backwards digit span. With regard to BNT total score, AD patients performed worse than both aMCI patients and controls (p<0.001), while aMCI subjects performed worse than controls on BNT spontaneous answers (without cues; p<0.05). The absolute values of spontaneous errors and total number of semantic errors are shown in Table 1. Regarding brain atrophy, results revealed a continuum among the groups, as shown in a previous study involving the same subjects.^27^

**Table 1:** Demographic and neuropsychological data.

	AD	aMCI	Controls	P
Age	74.26±6.33	68.29±9.93	69.12±7.55	0.170
Education	6.00±5.52	5.88±4.32	6.87±3.66	0.315
MMSE	22.93±2.65	26.41±2.76	29.12±0.71	<0.0001
BNT-total score (spontaneous + cued correct answers)	39.33±9.98	50.82±7.66	53.75±4.18	<0.0001
BNT- spontaneous answers	34.87±9.7	48.25±9.13	51.62±5.87	<0.05
Omission errors	6.43±5.39	2.50±2.65	1.62±2.50	0.006
Visual paragnosia	7.87±3.72	4.18±4.73	2.00±2.19	<0.0001
Semantic errors	10.31± 4.06	4.81±3.16	4.43±2.44	<0.0001
CAMCOG's Similarities	4.87±1.74	6.93±1.18	7.37±1.02	<0.001
A7-RAVLT	1.26±1.28	4.17±2.40	9.56±3.03	<0.0001
VF	10.60±3.39	13.64±3.92	19.43±3.03	<0.0001
VSP-LNI	17.20±1.42	18.76±0.97	18.81±0.98	0.002
fDS	4.46±1.06	4.58±0.79	5.06±0.85	0.108
bDS	3.20±0.77	3.11±0.92	4.12±1.02	0.004

Data expressed as mean±SD. MMSE: Mini-Mental State Examination; A7-RAVLT: delayed recall of Rey Auditory Verbal Learning Test; BNT: Boston Naming Test; VF: Verbal Fluency; VSP-LNI: visuospatial perception item of Luria's neuropsychological investigation; fDS: Forward Digit Span; bDS: backward Digit Span.

**Figure 1:**
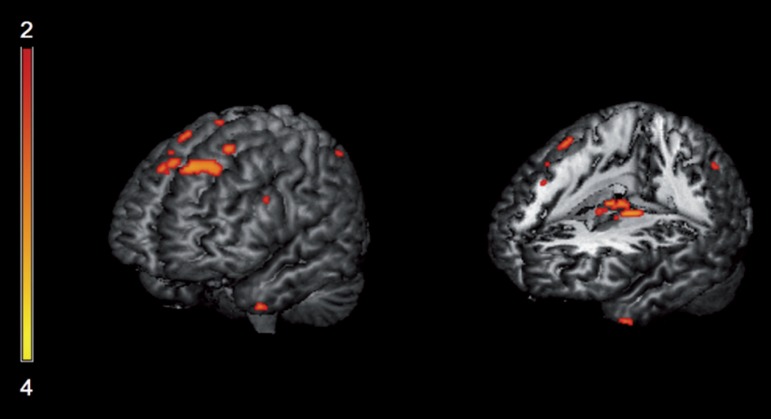
Areas exhibiting significant correlation with BNT total score, predominantly in left superior frontal girus, left inferior frontal gyrus, left anterior temporal pole and bilateral thalami (p<0.05, FWE corrected).

**Figure 2:**
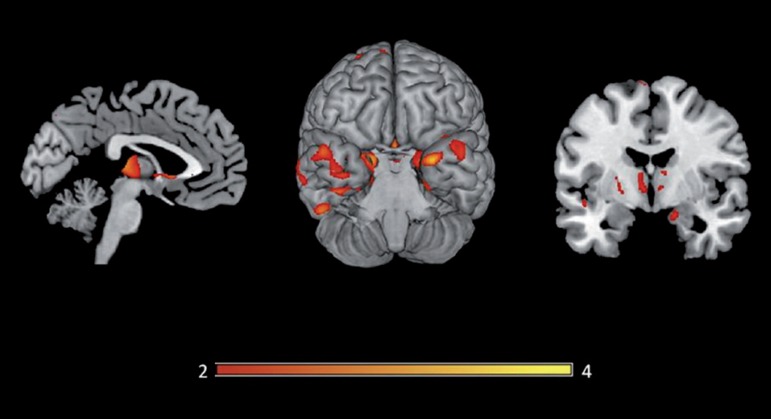
Areas exhibiting significant correlation with spontaneous semantic errors on BNT. All slices are in neurological orientation (left on the left side) p<0.05, FWE corrected.

Multiple regression analysis revealed significant correlations between GMD and BNT score, mostly in the thalamus: right lateral dorsal nucleus and left medial dorsal nucleus; bilateral hippocampus; bilateral parahippocampal gyrus; left superior temporal gyrus; left inferior frontal gyrus; bilateral superior frontal gyrus; left middle frontal gyrus, and other areas shown in Table 2 and Figure 1. Areas of correlations with spontaneous errors are shown in Table 3 and Figure 2. Semantic errors were related mainly to the bilateral anterior part of the temporal lobe: superior temporal gyrus; left inferior temporal gyrus; bilateral dorsomedial thalamic nucleus; bilateral hippocampal and left caudate nucleus.

**Table 2:** Brain areas exhibiting statistically significant correlation with BNT score.

Region	Number of voxels in cluster	Talairach coordinates			Z score
Left parahippocampal gyrus	97	-28	-40	-5	2.82
Left hippocampus	79	-29	-15	-18	2.35
Right hippocampus	80	34	-12	-20	2.11
Right thalamus, pulvinar	87	9	-28	9	2.35
Left thalamus, ventral anterior nucleus	99	-12	-7	13	2.17
Left thalamus, lateral dorsal nucleus	95	-10	-17	17	2.09
Left inferior frontal gyrus (BA 44)	73	-50	5	19	2.02
Left superior frontal gyrus (BA 9)	84	-1	55	25	2.07
Left precuneus	96	-17	83	40	2.00
Left superior frontal gyrus (BA 8)	77	-17	36	51	3.05
Right superior frontal gyrus (BA 6)	79	17	26	58	2.66

BA: Brodmann's area.

**Table 3:** Brain areas exhibiting greatest statistically significant correlation with semantic errors.

Region	Number of voxels in cluster	Talairach coordinates			Z score
Left superior temporal gyrus (BA 38)	100	-29	6	-28	4.32
Right superior temporal gyrus (BA 38)	88	38	10	-28	3.45
Right middle temporal gyrus (BA 21)	109	44	3	-34	3.48
Left middle temporal gyrus (BA 21)	72	-48	1	-21	2.90
Left inferior temporal gyrus (BA 20)	119	-49	-4	-37	2.21
Right inferior temporal gyrus (BA 20)	83	45	-12	-37	3.16
Right parahippocampal gyrus (BA 28)	60	16	-3	-13	2.48
Left uncus	54	-21	-7	-37	3.00
Left globus pallidus	103	-21	-3	-6	2.67
Right anterior cingulate (BA 25)	51	1	10	-3	2.33
Left thalamus, medial dorsal nucleus	55	-1	19	10	2.70
Right thalamus, medial dorsal nucleus	87	3	-20	6	2.39
Left thalamus, lateral dorsal nucleus	95	-11	-19	14	2.44
Right thalamus, lateral dorsal nucleus	95	11	-19	14	2.09
Right caudate nucleus	84	9	17	1	2.40
Left caudate nucleus	86	-6	4	1	2.36
Left putamen	104	-23	-2	1	2.81
Left inferior frontal gyrus (BA 44)	91	-51	9	20	2.16
Right inferior frontal gyrus (BA 44)	83	52	4	20	2.12
Left precuneus (BA 7)	101	-3	-76	44	2.73

BA: Brodmann's area.

## Discussion

Our results support the hypothesis of a continuum in brain pathology and cognitive decline among the three groups, particularly regarding their spontaneous answers during BNT picture naming, which indicates that our lesion model was satisfactorily tested. Several brain regions were found to be negatively correlated with semantic errors on the BNT (i.e. the more errors made, the lower the GMD in that particular area), and positively correlated with BNT score. A discussion follows on the possible role of each of these areas in the lexical-semantic networks and their contribution to the models of semantic memory organization.

Medial temporal structures such as the hippocampus and parahippocampal gyrus have a well-known role in episodic memory processes. Recently, they have also been associated with lexical-semantic memory. In fact, episodic and semantic memories are highly interactive.^28^ It is well established that episodic memory for events encoded during semantic categorization is better remembered than when subjects do not associate the target event with a particular previously learned characteristic, which indicates a close relationship between semantic and episodic memories. It is also possible that, through repetition and rehearsal, new information could be abstracted from its episodic context and represented as semantic memory.^29^ In addition, it has been demonstrated that amnesic patients with lesions in the medial temporal lobes have impaired acquisition of new semantic memories.^30^ Our results concur with those of a recent VBM study in patients with early AD,^31^ which also found strong GMD correlations in the medial temporal structures with naming performance, mainly with the most anterior part of the parahippocampus and other parts of the perirhinal cortex. As proposed by these authors, the primary role of this region is combining the different representations of a given object, as part of a process of multimodal synthesis spread across different cortical areas. Thus, lesion of these brain structures in early AD would isolate the hippocampus from the multisensory input of the neocortex, resulting in reduction of retrieval efficiency, rather than loss of representation.

The role of the thalamus in lexical-semantic memory is less well understood than that of other significant areas demonstrated in our study. Recent electrophysiological and functional neuroimaging studies have established the involvement of the thalamus in the process of feature binding, which results in the recall of the object in semantic memory.^31^ Researchers have proposed that the thalamus could modulate the mechanism for semantic object recall via synchronizing of electrical brain rhythms.^32^ Kraut et al. (2003)^33^ studied a word-word feature-binding task using event-related fMRI. They found two distinct loci of thalamic signal change, one anterior in the dorsomedial nucleus, and the other posterior in the pulvinar. Based on these findings and previous electrophysiological studies, the authors proposed a neural mechanism in which the dorsomedial nucleus is involved in the early search or object generation and activates other prefrontal regions specifically involved in task-related working memory or language functions. Our findings support the idea that the thalamus is directly involved in lexical-semantic memory activities, possibly with an integrative role, since its nuclei were correlated with both BNT total score and semantic errors.

The involvement of neocortical temporal regions in semantic memory is better understood and has been extensively demonstrated.^34-37^ Grossman et al. (2004)^37^ studied VBM and confrontation naming in AD, frontotemporal dementia, and corticobasal degeneration, and found a left lateral temporal atrophy as a common source of impaired naming across these patient groups. Another VBM study of semantic dementia^38^ showed that ATL activation peaks aligned closely with areas of strongest grey matter reduction, mostly with atrophy of the left anterior temporal lobe. We found correlations, particularly in the anterior parts of the STG, bilaterally but stronger on the left side, and in the anterior parts of the ITG, albeit weaker and less spread out than in the STG. Our findings support the notion that the anterior temporal lobe (ATL), predominantly its superior part, is strongly related to semantic generalization, since the subjects were asked to name pictures of different categories and a close relationship was observed between semantic errors, regardless of their specific categories.

The prefrontal cortex is also related to the lexical-semantic system, often in an asymmetrical manner, with the left side more involved than the right. The left inferior prefrontal cortex (LIPFC) has been regarded as a “semantic working memory system” responsible for retrieving, maintaining, monitoring, and manipulating semantic representations stored elsewhere,^6^ as evidenced by functional neuroimaging, transcranial magnetic stimulation, and lesion studies.^39-41^ On functional imaging studies, the LIPFC is more active when subjects make semantic judgments regarding words than when they make non-semantic judgments for the same words,^39^ and also when they make semantic judgments for line drawings.^42^ The role of the LIPFC is crucial when the semantic tasks require cognitive control of semantic or lexical retrieval, particularly during selection among competing alternatives. A study suggests that the LIPFC does not support retrieval of semantic knowledge per se.^43^ Rather, this retrieval is done entirely by the posterior neocortex based upon cues presented through bottom-up processes, and the specific role of the LIPFC would be to select those retrieved representations that are task-relevant from among competing, irrelevant representations.

Patients with left prefrontal lesions often have difficulty retrieving words in response to specific cues (e.g. words beginning with a specific letter or names of objects belonging to a specific semantic category), even when there is no aphasia.^44^ In such cases, making a semantic error (for example, naming “animal” instead of the target word “dog”) might indicate difficulty in selecting the appropriate phonological response to answer a particular semantic question. In fact, activation of the LIPFC has been elicited by phonological tasks such as discrimination of visually and auditorily presented words^45^ with the greatest activation more posteriorly near Broca’s area.^39^ These and other studies^46^ have also suggested a domain-specificity of the anterior LIPFC (BA 45/47) for controlled semantics and of the posterior LIPFC (BA 44/6) for controlled phonology. However, more recent studies^47,48^ have argued against domain-specificity and for domain-preferentiality in LIPFC. Thus, it may be hypothesized that the LIPFC is activated to the extent that lexical and semantic information must be rehearsed, temporarily stored, and selected in working memory to perform a particular task.

Our study has some limitations including the relatively small sample size and the fact that the BNT is not well balanced in terms of psycholinguistic variables. Despite this factor, the BNT is one of the most widely used naming tests in clinical practice, and it continues to be a well-accepted measure of naming impairment in brain-damaged patients. Notwithstanding the limitations of this study, we found evidence that several brain areas are related to the process of higher-order semantic generalization, particularly the thalamus, medial temporal lobe, prefrontal cortex (left more than right), and bilateral anterior temporal lobes (predominantly STG and ITG).

## References

[1] Luria AR (1986). The word and its semantic structure. Thought and language: the Luria's last conferences.

[2] McClelland JL, Rumelhart DE (1985). Distributed memory and the representation of general and specific
information. J Exp Psychol Gen.

[3] Hart Jr, Kraut MA, Hart Jr J, Kraut MA (2007). Neural hybrid model of semantic object memory (version
1.1). Neural basis of semantic memory.

[4] Hodges JR, Graham N, Patterson K (1995). Charting the progression in semantic dementia: implications for
the organisation of semantic memory. Memory.

[5] Patterson K, Nestor PJ, Rogers TT (2007). Where do you know what you know: The representation of semantic
knowledge in the human brain. Nat Rev Neurosci.

[6] Martin A, Chao LL (2001). Semantic memory and the brain: structure and
processes. Curr Opin Neurobiol.

[7] Albert MS, Heller HS, Milberg W (1988). Changes in naming ability with age. Psychol Aging.

[8] LaBarge E, Edwards D, Knesevich JW (1986). Performance of normal elderly on the Boston Naming
Test. Brain Lang.

[9] Zec RF, Markwell SJ, Burkett NR, Larsen DL (2005). A longitudinal study of confrontation naming in the "normal"
elderly. J Int Neuropsychol Soc.

[10] Adlam AL, Bozeat S, Arnold R, Watson P, Hodges JR (2006). Semantic knowledge in mild cognitive impairment and mild
Alzheimer's disease. Cortex.

[11] Dudas RB, Clague F, Thompson SA, Graham KS, Hodges JR (2005). Episodic and semantic memory in mild cognitive
impairment. Neuropsychologia.

[12] Winblad B, Palmer K, Kivipelto M (2004). Mild cognitive impairment--beyond controversies, towards a
consensus: report of the International Working Group on Mild Cognitive
Impairment. J Int Med.

[13] Kaplan EF, Goodglass H, Weintraub S (1983). The Boston Naming Test.

[14] Morris JC (1993). The Clinical Dementia Rating (CDR): current version and scoring
rules. Neurology.

[15] McKhann G, Drachman D, Folstein M, Katzman R, Price D, Stadlan EM (1984). Clinical diagnosis of Alzheimer's disease: report of the
NINCDS-ADRDA Work Group under the auspices of Department of Health and Human
Services Task Force on Alzheimer's Disease. Neurology.

[16] Folstein MF, Folstein SE, McHugh PR (1975). "Mini-mental state": a practical method for grading the cognitive
state of patients for the clinician. J Psychiatr Res.

[17] Brucki SM, Nitrini R, Caramelli P, Bertolucci PH, Okamoto IH (2003). Suggestions for utilization of the mini-mental state examination
in Brazil. Arq Neuropsiquiatr.

[18] Rey A (1964). Clinical examination in psychology.

[19] Roth M, Huppert FA, Tym E, Mountjoy CQ (1988). CAMDEX: The Cambridge Examination for Mental Disorders of the
Elderly.

[20] Christensen A-L (1979). Luria's Neuropsychological Investigation.

[21] Wechsler D (1987). Manual for the Wechsler Memory Scale-Revised (WMS-R).

[22] Alexopoulos GS, Abrams RC, Young RC, Shamoian CA (1988). Cornell Scale for Depression in Dementia. Biological Psychiatry.

[23] Carthery-Goulart MT, Areza-Fegyveres R, Schultz RR (2007). Brazilian version of the Cornell depression scale in
dementia. Arq Neuropsiquiatr.

[24] Ashburner J, Friston KJ (2005). Unified segmentation. NeuroImage.

[25] Bergougnian L, Chupin M, Czechowska Y (2009). Can voxel based morphometry, manual segmentation and automated
segmentation equally detect hippocampal volume differences in acute
depression?. Neuroimage.

[26] Rorden C, Fridriksson J, Karnath HO (2009). An evaluation of traditional and novel tools for lesion behavior
mapping. Neuroimage.

[27] Balthazar ML, Cendes F, Damasceno BP (2008). Semantic error patterns on the Boston Naming Test in normal
aging, amnestic mild cognitive impairment and mild Alzheimer's disease: is
there semantic disruption?. Neuropsychology.

[28] Tulving E (1987). Multiple memory systems and consciousness. Hum Neurobiol.

[29] Squire LR, Knowlton B, Musen G (1993). The structure and organization of memory. Ann Rev Psychol.

[30] Gabrieli JD, Cohen NJ, Corkin S (1988). The impaired learning of semantic knowledge following bilateral
medial temporal-lobe resection. Brain Cog.

[31] Venneri A, McGeown WJ, Hietanen HM, Guerrini C, Ellis AW, Shanks MF (2008). The anatomical bases of semantic retrieval deficits in early
Alzheimer's disease. Neuropsychologia.

[32] Slotnick SD, Moo LR, Kraut MA, Lesser RP, Hart J Jr (2002). Interactions between thalamic and cortical rhythms during
semantic memory recall in human. Proc Natl Acad Sci USA.

[33] Kraut MA, Calhoun V, Pitcock JA, Cusick C, Hart J Jr (2003). Neural hybrid model of semantic object memory: implications from
event-related timing using fMRI. J Int Neuropsychol Soc.

[34] Damasio H, Grabowski TJ, Tranel D, Hichwa RD, Damasio AR (1996). A neural basis for lexical retrieval. Nature.

[35] Gorno-Tempini ML, Price CJ, Josephs O (1998). The neural systems sustaining face and proper-name
processing. Brain.

[36] Damasio H, Tranel D, Grabowski T, Adolphs R, Damasio A (2004). Neural systems behind word and concept retrieval. Cognition.

[37] Grossman M, McMillan C, Moore P (2004). What's in a name: voxel-based morphometric analyses of MRI and
naming difficulty in Alzheimer's disease, frontotemporal dementia and
corticobasal degeneration. Brain.

[38] Mummery CJ, Patterson K, Price CJ, Ashburner J, Frackowiak RS, Hodges JR (2000). A voxel-based morphometry study of semantic dementia:
relationship between temporal lobe atrophy and semantic
memory. Ann Neurol.

[39] Gabrieli JD, Cohen NJ, Corkin S (1988). The impaired learning of semantic knowledge following bilateral
medial temporal-lobe resection. Brain Cog.

[40] Devlin JT, Russell RP, Davis MH (2002). Is there an anatomical basis for category-specificity? Semantic
memory studies in PET and fMRI. Neuropsychologia.

[41] Thompson-Schill SL, Swick D, Farah MJ, D'Esposito M, Kan IP, Knight RT (1998). Verb generation in patients with focal frontal lesions: a
neuropsychological test of neuroimaging findings. Proc Natl Acad Sci USA.

[42] Vandenberghe R, Price C, Wise R, Josephs O, Frackowiak RS (1996). Functional anatomy of a common semantic system for words and
pictures. Nature.

[43] Thompson-Schill SL, D'Esposito M, Aguirre DK, Farah MJ (1997). Role of left inferior prefrontal cortex in retrieval of semantic
knowledge: a re-evaluation. Proc Natl Acad Sci USA.

[44] Baldo JV, Shimamura AP (1998). Letter and category fluency in patients with frontal lobe
lesions. Neuropsychology.

[45] Fiez JA, Raichle ME, Miezin FM, Pettersen SE, Tallal P, Katz WF (1995). PET studies of auditory and phonological processing: Effects of
stimulus characteristics and task design. J Cog Neurosc.

[46] Poldrack RA, Wagner AD, Prull MW, Desmond JE, Glover GH, Gabrieli JD (1999). Functional specialization for semantic and phonological
processing in the left inferior prefrontal cortex. Neuroimage.

[47] Gold BT, Balota DA, Kirchhoff BA, Buckner RL (2005). Common and dissociable activation patterns associated with
controlled semantic and phonological processing: evidence from FMRI
adaptation. Cereb Cortex.

[48] Snyder HR, Feigenson K, Thompson-Schill SL (2007). Prefrontal cortical response to conflict during semantic and
phonological tasks. J Cog Neurosci.

